# Paeonol Attenuated Vascular Fibrosis Through Regulating Treg/Th17 Balance in a Gut Microbiota-Dependent Manner

**DOI:** 10.3389/fphar.2021.765482

**Published:** 2021-11-22

**Authors:** Xiaoyan Shi, Hanwen Huang, Min Zhou, Yarong Liu, Hongfei Wu, Min Dai

**Affiliations:** ^1^ College of Pharmacy, Anhui University of Chinese Medicine, Hefei, China; ^2^ Anhui Key Laboratory for Research and Development of Traditional Chinese Medicine, Hefei, China

**Keywords:** paeonol, atherosclerosis, gut microbiota, Treg/Th17 balance, vascular fibrosis

## Abstract

**Background:** Paeonol (Pae) is a natural phenolic compound isolated from Cortex Moutan, which exhibits anti-atherosclerosis (AS) effects. Our previous work demonstrated that gut microbiota plays an important role during AS treatment as it affects the efficacy of Pae. However, the mechanism of Pae in protecting against vascular fibrosis as related to gut microbiota has yet to be elucidated.

**Objective:** To investigate the antifibrosis effect of Pae on AS mice and demonstrate the underlying gut microbiota-dependent mechanism.

**Methods:** ApoE^-/-^ mice were fed with high-fat diet (HFD) to replicate the AS model. H&E and Masson staining were used to observe the plaque formation and collagen deposition. Short-chain fatty acid (SCFA) production was analyzed through LC-MS/MS. The frequency of immune cells in spleen was phenotyped by flow cytometry. The mRNA expression of aortic inflammatory cytokines was detected by qRT-PCR. The protein expression of LOX and fibrosis-related indicators were examined by western blot.

**Results:** Pae restricted the development of AS and collagen deposition. Notably, the antifibrosis effect of Pae was achieved by regulating the gut microbiota. LC-MS/MS data indicated that the level of SCFAs was increased in caecum contents. Additionally, Pae administration selectively upregulated the frequency of regulatory T (Treg) cells as well as downregulated the ratio of T helper type 17 (Th17) cells in the spleen of AS mice, improving the Treg/Th17 balance. In addition, as expected, Pae intervention can significantly downregulate the levels of proinflammatory cytokines IL-1β, IL-6, TNF-α, and IL-17 in the aorta, and upregulate the levels of anti-inflammatory factor IL-10, a marker of Treg cells. Finally, Pae’s intervention in the gut microbiota resulted in the restoration of the balance of Treg/Th17, which indirectly downregulated the protein expression level of LOX and fibrosis-related indicators (MMP-2/9 and collagen I/III).

**Conclusion:** Pae attenuated vascular fibrosis in a gut microbiota-dependent manner. The underlying protective mechanism was associated with the improved Treg/Th17 balance in spleen mediated through the increased microbiota-derived SCFA production. Collectively, our results demonstrated the role of Pae as a potential gut microbiota modulator to prevent and treat AS.

## Introduction

Atherosclerosis (AS) is a chronic inflammatory disease that is characterized by the development of fibrotic plaques in the arterial wall (Lan et al., 2013). Despite therapeutic advances in the management of chronic vascular inflammation (Sanchez-Madrid and Sessa, 2010; Colmorten et al., 2019), there have been limited improvements in specific antifibrotic therapies for AS. Gut microbiota and abnormal immune response have been implicated as the main risk factors for developing AS (Jonsson and Backhed, 2017). The development of AS plaques can be affected by a distant infection (Shah, 2019).

Recent interest has emerged in understanding the crosstalk between gut microbiota and the host immune system by controlling the balance of T (Treg) and T helper type 17 (Th17) cells (He et al., 2020; Saigusa et al., 2020). Th17 cells, characterized by the production of proinflammatory cytokines IL-17, are a major contributor in the development of AS (Taleb et al., 2015). In contrast, Treg cells have been identified as dedicated suppressors of diverse immune responses and inflammations through the secretion of the anti-inflammatory cytokine IL-10 (Ou et al., 2018). Th17 and Treg cells play an important role in regulating vascular plaque ECM composition and vascular fibrosis through modulating the expression of collagen and MMPs (Slobodin and Rimar, 2017). However, the reduced diversity of gut microbiota causes the Treg/Th17 balance to tend toward Th17 cells, which, in turn, aggravates vascular fibrosis.

Short-chain fatty acids (SCFAs) have been shown to promote Treg cell differentiation and inhibit inflammatory reaction (Liu et al., 2020). Therefore, gut microbiota diversity and microbiota-derived SCFAs altering the Treg/Th17 balance play important roles in the vascular fibrosis. The enzyme lysyl oxidase (LOX) upregulation is associated with vascular fibrosis (Martinez-Gonzalez et al., 2019). A study has shown that Th17 and Treg might modulate LOX expression through their characteristic cytokines IL-17 and IL-10, respectively, thereby regulating fibrosis (Lu et al., 2020). Taken together, the disruption of Treg/Th17 balance and gut microbiota-derived beneficial SCFA reduction are important parameters of the vascular fibrosis in AS.

Paeonol (2'-hydroxy-4'-methoxyacetophenone, Pae) is an active compound isolated from Cortex Moutan root, which has shown potent anti-AS, anti-inflammation, and even antibacterial biological activities (Pan and Dai, 2009; Wu et al., 2020). As a natural compound, the anti-AS activity of Pae has been suggested to be due to its anti-inflammation effect, which is related to its reduction of the production of NLRP3 inflammasomes in endothelial cells (Li et al., 2009; Shi et al., 2020). Furthermore, Pae has antifibrotic functions against pulmonary and renal fibrosis in mice, respectively (Liu et al., 2017; Zhang et al., 2018). Our previous work demonstrated that gut microbiota plays an important role during AS treatment as it affects the efficacy of Pae. However, the mechanism of Pae in protecting against vascular fibrosis as related to gut microbiota has yet to be elucidated.

In this study, we investigated for the first time the effect of Pae administration on vascular fibrosis. To identify the role of gut microbiota in mediating the protective effect of Pae on vascular fibrosis, gut microbiota depletion and fecal microbiota transplant (FMT) were conducted. Finally, the underlying gut microbiota-dependent mechanism was also explored.

## Materials and Methods

### Animals

ApoE^-/-^ mice weighing 17–27 g were purchased from Cavens Laboratory Animal Co., Ltd (Changzhou, China) and used to build the AS animal model. All male ApoE^-/-^ mice of 6 weeks of age were kept under pathogen-free conditions with 12/12 h light/dark cycle and fed with a high-fat diet (HFD, containing 21% fat and 0.15% cholesterol) until AS lesions were obviously formed on the arteries. Subsequently, AS animals were orally administrated with 200 and 400 mg/kg of Pae in contained 5% CMC-Na solution daily for a total of 4 weeks. At the same time, 6-week-old male C57BL/6 mice received water during the experiment as the control group.

### Chemicals and Reagents

Pae (99%purity) was obtained from Baicao Plants Biotech Co., Ltd. (Anhui, China). Antibodies for FACS included PerCP-Cy5.5-labeled antimouse CD4 antibody (BD, Cat. NO. 550954), APC-labeled antimouse IL-17A antibody (BD, Cat. NO. 506915), BV421-labeled antimouse CD25 antibody (BD, Cat. NO. 564370), and PE-labeled Foxp3 Monoclonal Antibody (ebioscience, Cat. NO. 12-5773-80). Total SCFA ELISA kits were obtained from Multi Science Biotechnology Co., Ltd. (Hangzhou, China).

### Hematoxylin and Eosin Staining

The arteries were placed in 4% paraformaldehyde for 24 h and then transferred to 70% ethanol. Next, the arteries were processed to paraffin-embedded blocks to generate 5-µm-thick sections for H&E staining. Subsequently, the sections were stained with hematoxylin and eosin. After dehydration, resinene was used to seal the sections to transparency. The analysis was performed by an inverted microscope.

### Masson Staining

Masson staining was used to detect collagen fibers in the plaque of aortic vessels. According to the kit for Masson staining manufacturer instructions, the aortic vessels fixed in 4% paraformaldehyde were dehydrated in alcohol, paraffin-embedded, sectioned, and subjected to Masson staining. The stained sections were observed under a light microscope.

### Antibiotic Treatment and Fecal Microbiota Transplantation

Six-week-old male C57BL/6 J mice fed with an HFD were supplemented with either 0.5% CMC-Na (solvent) or Pae at 400 mg/kg in the absence or presence of antibiotics [vancomycin (0.5 g/L), neomycin sulfate (1 g/L), metronidazole (1 g/L), and ampicillin (1 g/L)]. Fecal transplant was performed on the basis of our preliminary research protocol. Briefly, 6-week-old male donor mice were fed with HFD or HFD + Pae (400 mg/kg). The recipient mice were fed with HFD and treated daily with fresh transplant material from either Pae-treated mice or HFD mice. Fresh fecal from Pae-treated mice or HFD mice were collected and diluted immediately with sterile saline (100 mg/ml). The solution was vigorously mixed, centrifuged at 8,000 rpm, for 5 min to get the total bacteria. Finally, the supernatant was obtained and used as transplant material.

### Quantification of Caecum SCFAs by LC-MS/MS

The level of SCFAs was measured with LC-MS/MS analysis. Caecum contents were diluted with nanopure water and centrifuged at 12,000 rpm for 10 min. The supernatant was filtered through a 0.45-μm microfiltration membrane. Then, the filtered supernatant was mixed with an 185-μl mixed extract (80% acetonitrile and 20% methanol) and centrifuged at 18,000 rpm for 20 min. Subsequently, the supernatant was derivatized and then added to the internal standard for LC-MS/MS analysis.

### Flow Cytometry Analysis

At the end of the experiment, spleen tissues of ApoE^-/-^ mice were collected and then single-spleen cell suspension was immediately prepared for flow cytometry analysis. Single-spleen cells were first stimulated with 1.5 μl of a stimulation inhibitor and cultured for 7 h in 5% CO_2_ at 37°C. Subsequently, cells were stained with live/dead dye and surface markers for 30 min at 4°Cin the dark, and then the cells were fixed and permeabilized with fixation/permeabilization working solution and permeabilization buffer for 20 min at RT in the dark. Finally, cells were stained with the anti-IL-10 or anti-IL-17A antibody. Flow cytometric analysis was used to detect the Th17 and Treg cells with CD4-PerCP-Cy5.5, IL-17A-APC antibodies and CD25-BV421, FOXP3-PE antibodies, respectively.

### RNA Isolation and Real-Time Reverse Transcription Quantitative Polymerase Chain Reaction

Total RNA was isolated from mice aortic vessels using Trizol, and first-strand cDNA was synthesized from 1 µg of total RNA according to the manufacturer’s instructions. RT-qPCR was used to detect the expression of β-actin, IL-1β, IL-6, TNF-α, IL-10, and IL-17. Relative expression was evaluated by the comparative CT method and normalized to the expression of U6 small RNA. Changes in the gene expression were determined using the 2^-∆∆Ct^ method.

### Western Blotting

Total proteins from aortic tissues were extracted using a RIPA lysis buffer (Beyotime, Haimen, China) and quantified by a BCA Protein Assay kit. Protein samples were electrophoresed using 10% polyacrylamide gels and transferred to 0.45-µm PVDF membranes (Merck Millipore, United States). After the membranes were blocked with 5 % BSA for 1 h at 37°C, the blots were probed with α-SMA antibody (Abcam, United States; 1:2,000), LOX antibody (ZENBIO, China; 1:1,000), MMP-2 antibody (ZENBIO, China; 1:1,000), MMP-9 antibody (ZENBIO, China; 1:1,000), collagen-I antibody (ZENBIO, China; 1:1,000), collagen-Ⅲ antibody (ZENBIO, China; 1:1,000), and GAPDH antibody (Sigma, United States; 1:1,000) throughout as loading controls. After primary antibody incubation, membranes were washed and incubated with HRP-conjugated secondary antibody. The protein signals were visualized by the enhanced chemoluminescence kit (Thermo, United States).

### Statistical Analysis

The data were presented as mean ± SD and analyzed using the SPSS 23.0 software. Differences between groups were analyzed by the Student’s *t*-test and one-way ANOVA. A value of *p* < 0.05 or *p* < 0.01 was considered to be statistically significant.

## Results

### Pae Restricted Plaque Formation and Vascular Fibrosis in ApoE^-/-^ Mice

**FIGURE 1:**
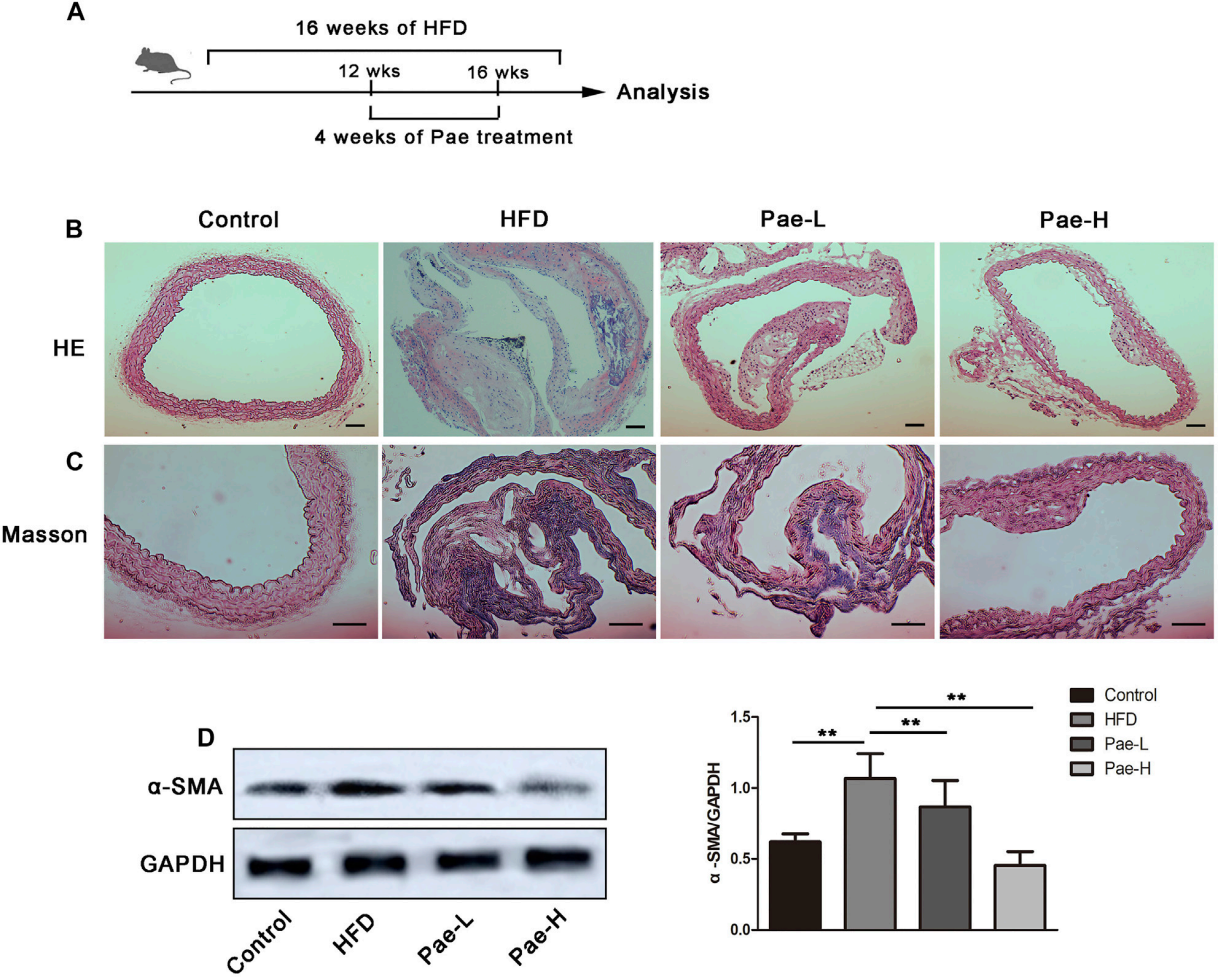
Pae restricted plaque formation and collagen deposition of aorta in ApoE^-/-^ mice. **(A)** Scheme of the experiment. ApoE^-/-^ mice were administered with Pae for 4 weeks starting at 12 weeks of HFD feeding. **(B, C)** H&E and Masson staining of aorta (scale bars, 100 μm). **(D)** α-SMA protein expression in the aorta by Western blot. Data are presented as mean ± SEM, *n* = 3. *p < 0.05, **p < 0.01.

To investigate the potential antifibrosis effect of Pae, we detected plaque formation and collagen deposition in the aorta of AS mice (Figure 1A). Pae was orally administered with 400, and 200 mg/kg body weight in a 5% CMC-Na solution daily for a total of 4 weeks. In the Pae-treated group, plaque size was moderately lower compared to the model group and according to the H&E staining (Figure 1B). Masson staining showed that Pae significantly reduced the collagen deposition in the aortic vessels (Figure 1C), and the protein expression level of α-SMA was significantly downregulated (Figure 1D), indicating that Pae treatment reduced vascular fibrosis and restricted AS lesion development in ApoE^-/-^ mice.

### Pae Alleviated Plaque Formation and Vascular Fibrosis in a Gut Microbiota-Dependent Manner

**FIGURE 2:**
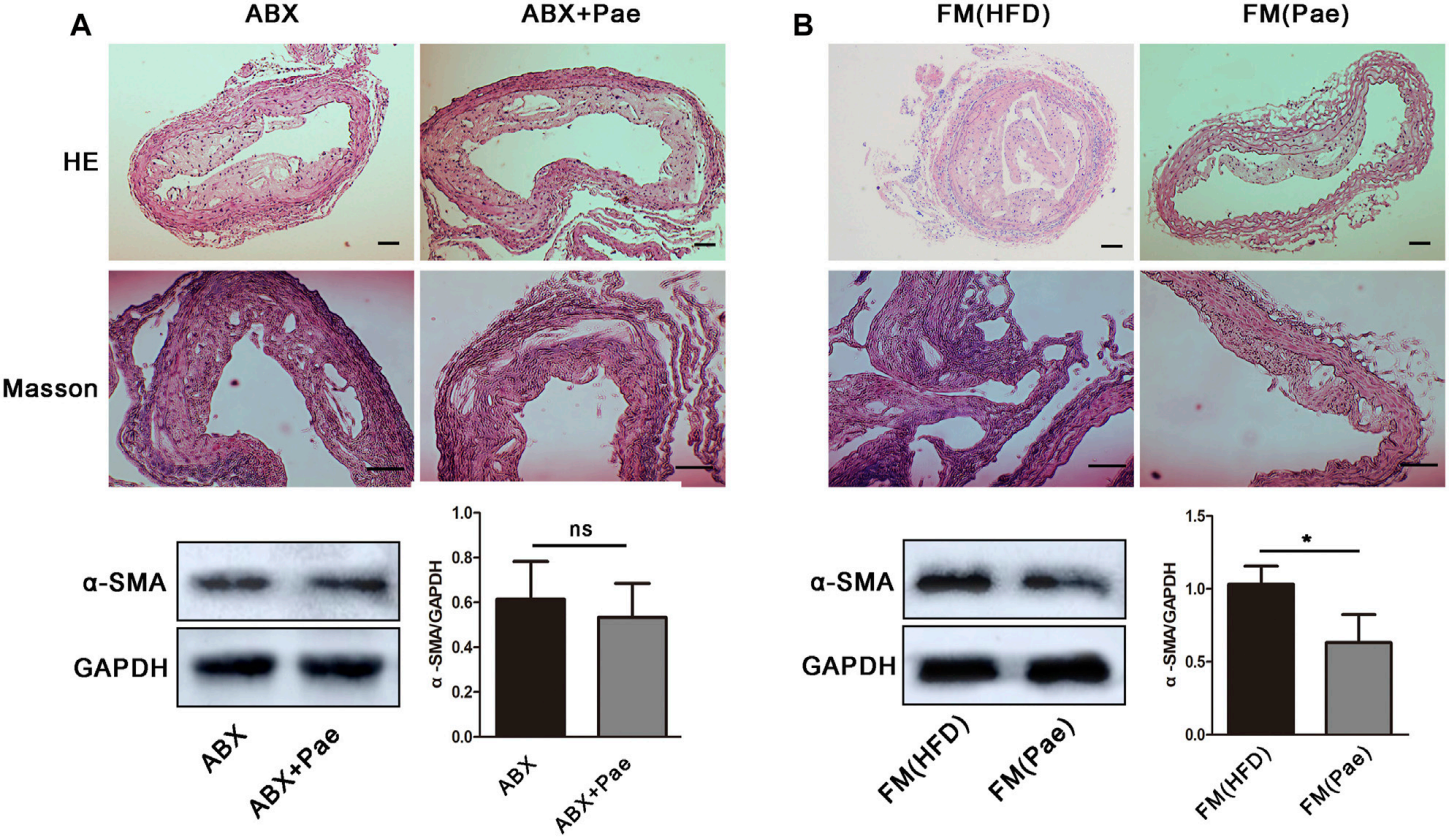
Microbiota drive the antifibrosis effect in Pae-treated ApoE^-/-^ mice. **(A)** The antifibrosis effect of Pae against HFD-induced AS disappeared after gut-microbiota depletion. **(B)** Pae microbial transplantation mitigated HFD-induced vascular fibrosis. Data are presented as mean ± SEM, *n* = 3. ^*^ *p* < 0.05, ^**^ *p* < 0.01.

Although the precise etiology and pathogenesis of AS remain unclear, abundant evidence has indicated that dysregulated responses to gut microbiota alteration contribute to the occurrence of AS, making the gut microbiota a new target for AS treatment. To investigate whether gut microbiota participated in the antifibrosis effect of Pae, ApoE^-/-^ mice were gavaged using the antibiotic cocktails for gut microbiota depletion (ABX group) while giving Pae to intervene together (ABX+Pae group). Our results demonstrated that there were no significant differences between plaque formation, collagen deposition, and α-SMA protein expression in the ABX group compared with the ABX+Pae group (Figure 2A).

To further confirm whether the antifibrosis effect in Pae-treated mice depends on gut microbiota, we performed FMT experiments in which gut microbiota-depleted ApoE^-/-^ mice were reconstituted with the microbiota of HFD-treated mice [FM(HFD)→ApoE^-/−^recipients, FM(HFD)] and Pae-treated mice [FM(Pae+HFD)→ApoE^-/−^recipients, FM(Pae)] *via* intragastric administration once a day for 4 weeks. For better microbiota colonization, all experimental mice were gavaged with the antibiotic cocktails to create gut microbiota depletion conditions before FMT. We found that microbiota transfer from Pae-treated donors significantly reduced the formation of plaque, collagen deposition, and α-SMA protein expression in aortas (Figure 2B), suggesting that a particular constellation of microbial compounds, available in Pae-treated mice, may be capable to influence the vascular fibrosis and AS development.

### Pae Treatment Increased Gut Microbiota-Derived SCFAs

Mounting evidence supports the proposal of a role for the microbiota in the progression of AS. Moreover, Pae ameliorated acute ALD-related inflammatory injury to the liver by regulating gut microbiota (Wu et al., 2020). To confirm which bacterium was altered by Pae treatment and, in turn, affected the disease progression against AS, our team studied the effect of Pae on the abundance and diversity of the gut microbiota in mice (unpublished data). Our previous research data showed that the relative abundance of SCFA-producing microbiota (Clostridia, *Clostridium* Ⅳ, Lachnospiraceae, and *Lactobacillus*) displayed a relatively low abundance in the HFD group, while the relative abundance was significantly enriched after Pae intervention. Together, these results demonstrated that Pae treatment effectively increased the numbers of SCFA-producing bacteria.

**FIGURE 3:**
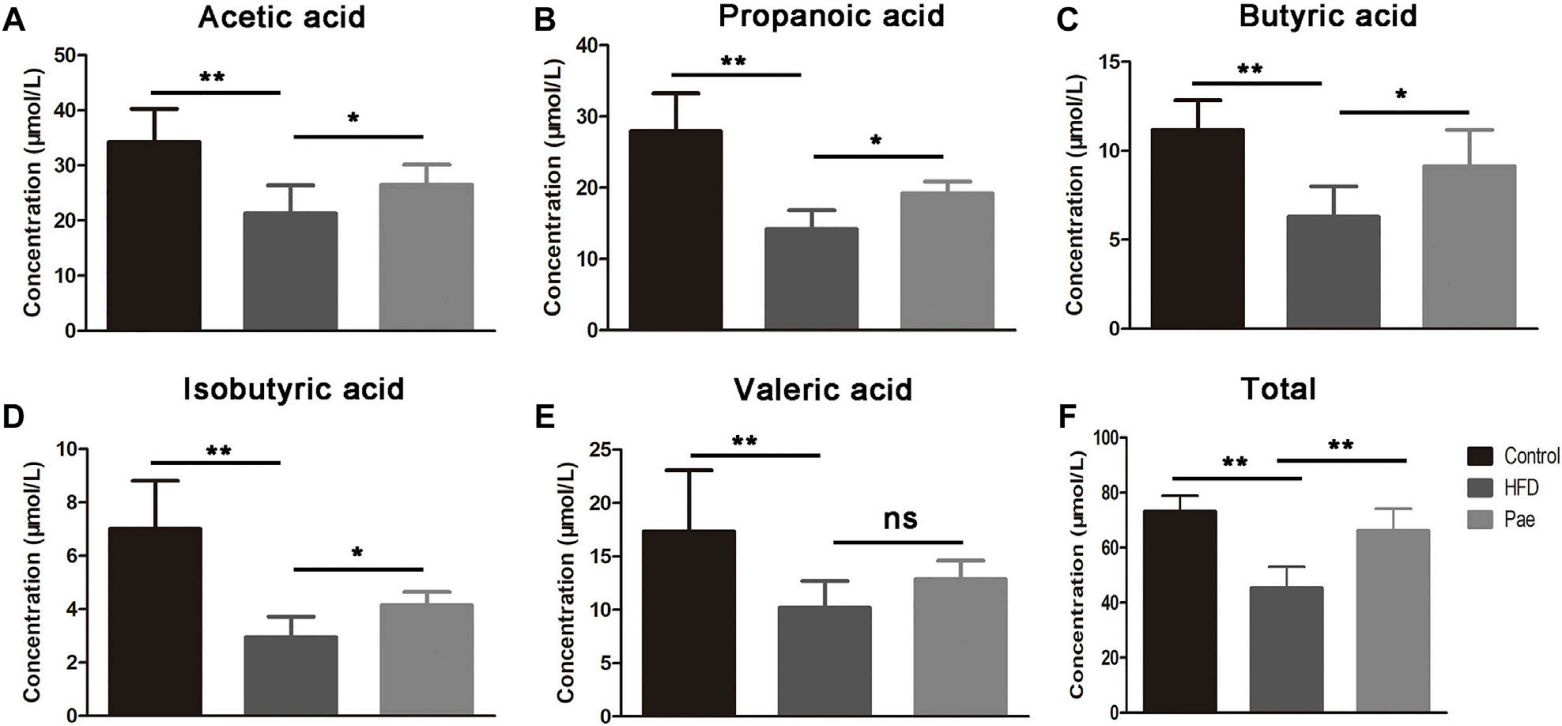
Pae treatment increased the production of microbial metabolites SCFAs. **(A–E)** SCFA concentration from cecal content. **(F)** Total SCFA concentration from serum. Data are presented as mean ± SEM, *n* = 6. *p < 0.05, **p < 0.01.

SCFAs are bacterial metabolites from dietary components, and SCFAs produced by commensal bacteria stimulate the generation of Treg cells. To investigate whether the changes in bacterial communities had an impact on the microbial metabolic output, we further detected the levels of several important SCFAs in the feces and total SCFA levels in the serum (Figure 3). Consistent with the changes in microbiota abundance, the Pae and HFD groups had totally different SCFA profiles. The Pae group manifested higher amounts of acetic acid, propionic acid, butyric acid, and isobutyric acid in the feces. Although there were no significant differences in terms of valeric acid levels, total SCFAs in the Pae group were significantly higher than those in the HFD group in the serum.

### Pae Improved Treg/Th17 Balance in AS Mice

**FIGURE 4:**
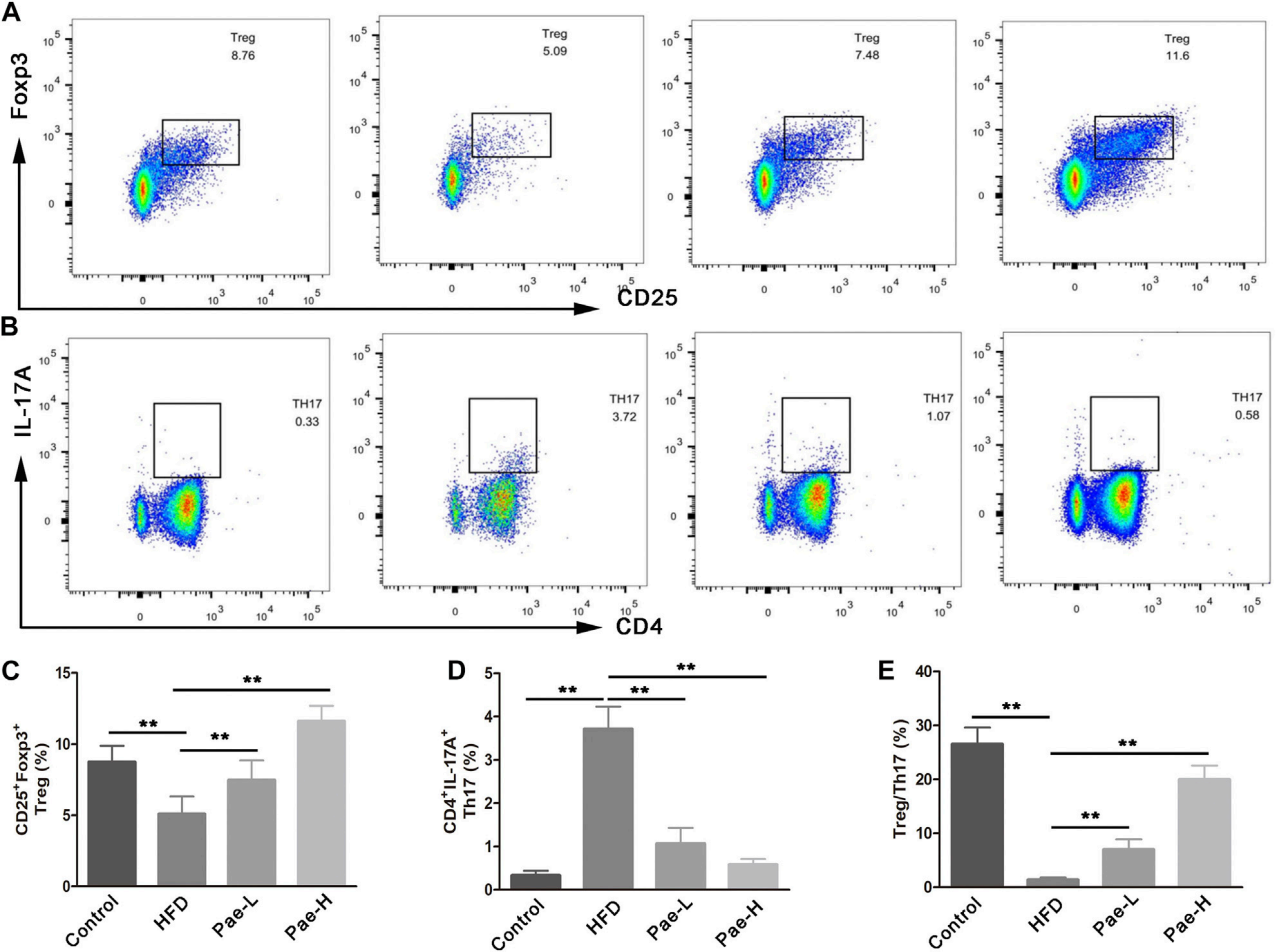
Pae improved Treg/Thl7 balance in HFD-induced AS model mice. **(A,C)** Flow cytometric analysis showed that Pae treatment increased the numbers of Treg (CD4^+^CD25^+^Foxp3^+^) cells and **(B,D)** decreased the numbers of Th17 (CD4^+^IL17A^+^) cells in spleen compared with the HFD group. Data are presented as mean ± SEM, *n* = 6. ^*^ *p* < 0.05, ^**^ *p* < 0.01.

Previous reports have shown that the imbalance of Th17 and Treg cells was critically involved in the development of AS, and that coordinated regulation of Th17 and Treg cells would improve the vascular inflammation microenvironment and confer therapeutic benefits for vascular fibrosis. Several reports pointed out that SCFAs regulated the balance of Th17/Treg cells. As mentioned above, Pae significantly increased the content of SCFAs in AS mice. To investigate whether Pae treatment exerted an influence on systemic immune responses, we investigated the impact of Pae administration on Treg/Th17 balance in AS mice. Specifically, T cell responses in the spleen tissue were phenotyped by flow cytometry. The relative abundance of Treg and Th17 cell type was reported as a percent of the overall CD4^+^ T cells. As shown in Figure 4, the ratio of CD4^+^IL17A^+^(Th17) cells in spleen lymphocytes was increased, and the proportion of CD4^+^CD25^+^Foxp3^+^(Treg) cells in spleen lymphocytes was decreased in the HFD group compared with the control group. Pae treatment decreased the number of CD4^+^IL17A^+^(Th17) cells and increased the number of CD4^+^CD25 + Foxp3^+^(Treg) cells in spleen lymphocytes. In summary, Pae administration selectively upregulated the frequency of Treg cells as well as downregulated the ratio of Th17 cells, improving the Treg/Th17 balance to maintain host immune homeostasis.

### Pae Regulated Treg/Th17 Balance in a Gut Microbiota-Dependent Manner

**FIGURE 5:**
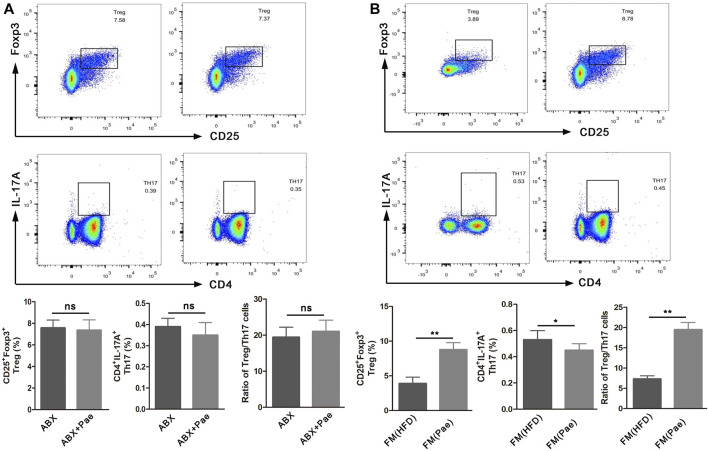
The frequency of Treg cells and Th17 cells in ApoE^-/-^ mice after gut microbiota depletion and FMT. **(A)** Representative plot and graph analysis of Treg (CD4^+^CD25^+^Foxp3^+^) cells and Th17 (CD4^+^IL17A^+^) cells in the spleen from ABX and ABX+Pae groups. **(B)** Representative plot and graph analysis of Treg (CD4^+^CD25^+^Foxp3^+^) cells and Th17 (CD4^+^IL17A^+^) cells in the spleen from FM(HFD) and FM(Pae) groups. Data are presented as mean ± SEM, *n* = 6. ^*^ *p* < 0.05, ^**^ *p* < 0.01.

To further investigate whether the improved Treg/Th17 balance after Pae administration was gut microbiota-dependent, spleen lymphocytes isolated from gut microbiota depletion groups were phenotyped by flow cytometry. The percentage of Treg cells (CD25^+^Foxp3^+^cells) and Th17 cells (CD4^+^IL17A^+^cells) displayed no significant difference between the ABX and ABX+Pae groups (Figure 5A).

To further confirm that Pae could improve the Treg/Th17 balance in AS mice by regulating gut microbiota, gut microbiota from HFD and Pae-treated mice were transferred into AS mice, respectively. Then, the numbers of Treg cells and Th17 cells in spleen lymphocytes were phenotyped in the FMT groups. As shown in Figure 5B, the number of CD4^+^IL17A^+^(Th17) cells in spleen lymphocytes was decreased in the FM(Pae) group as compared to the FM(HFD) group, whereas the proportion of CD25^+^Foxp3^+^(Treg) cells of spleen was increased in the FM(Pae) group as compared to the FM(HFD) group. These results indicated that the altered gut microbiota following Pae administration were responsible for the improved Treg/Th17 balance.

### Pae Administration Downregulated Proinflammatory Cytokines and Upregulated Anti-Inflammatory Cytokines in AS Mice

**FIGURE 6:**
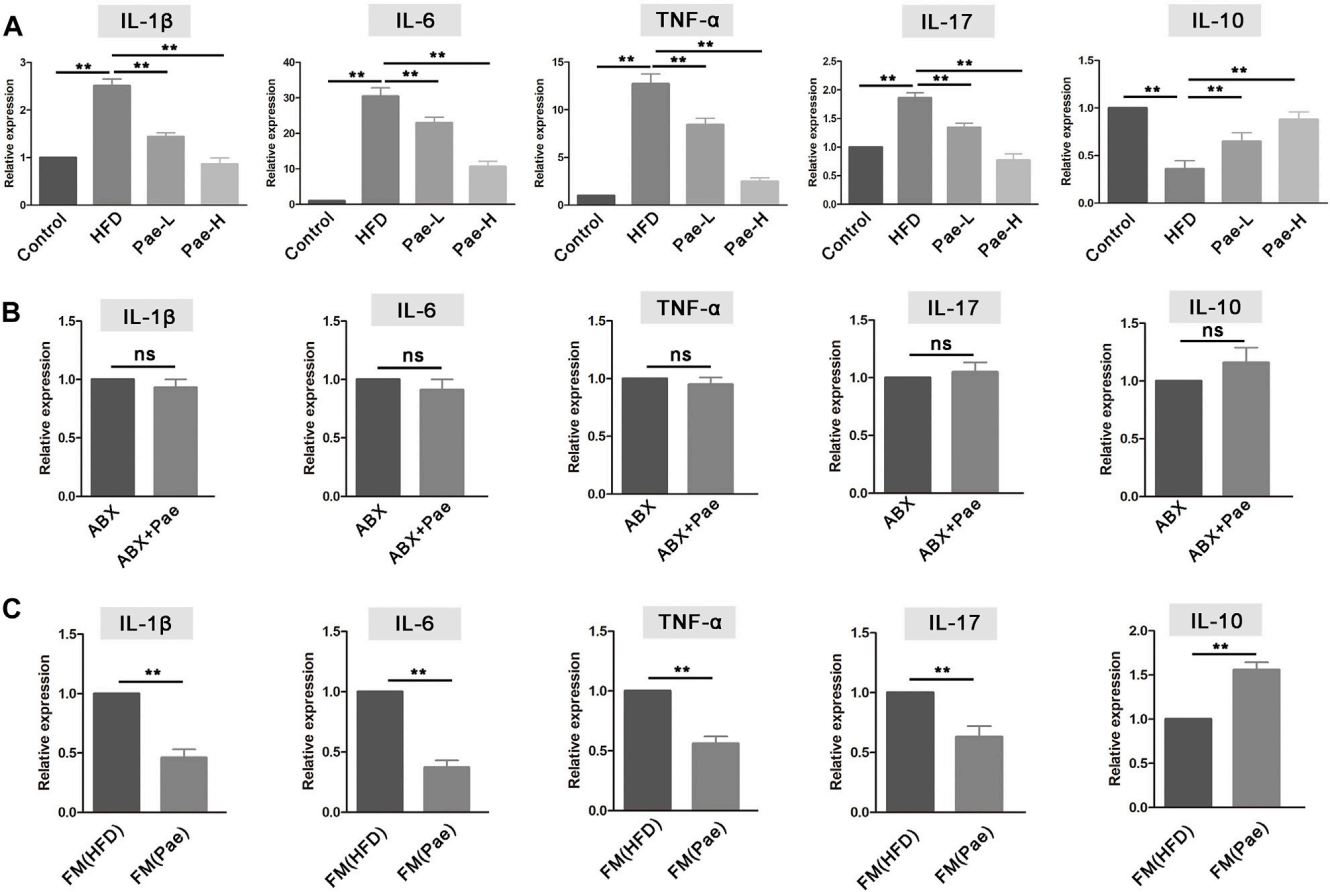
Pae treatment altered the level of mRNA expression inflammatory cytokine in ApoE^-/-^ mice. **(A)** Relative gene expressions of IL-1β, IL-6, TNF-α, IL-17, and IL-10 in aortas were measured by qRT-PCR in control, HFD, and Pae groups. **(B)** Relative gene expressions of IL-1β, IL-6, TNF-α, IL-17, and IL-10 in in aortas were measured by qRT-PCR between ABX and ABX+Pae groups. **(C)** Relative gene expressions of IL-1β, IL-6, TNF-α, IL-17, and IL-10 in aortas were measured by qRT-PCR between FM(HFD) and FM(Pae) groups. Data are presented as mean ± SEM, *n* = 6. ^*^ *p* < 0.05, ^**^ *p* < 0.01.

Immune inflammation plays an important role in the occurrence and development of AS. IL-10 is the key cytokine in Treg-mediated inflammatory suppression, while IL-17A is the Th17-mediated proinflammatory cytokine. Given the markedly altered Treg/Th17 balance after Pae administration, we further examined the expression levels of inflammatory cytokines in aortic tissues. Compared with the HFD group, the proinflammatory cytokines, including IL-1β, IL-6, TNF-α, and IL-17A, manifested a significantly decreasing trend in the Pae group. Instead, the anti-inflammatory cytokines IL-10 were significantly increased in the Pae group (Figure 6A).

The mRNA expression level of inflammatory cytokines in aortic tissues also conducted in gut microbiota-depletion groups. The levels of proinflammatory cytokines (including IL-1β, IL-6, TNF-α, and IL-17A) and the anti-inflammatory cytokine IL-10 exhibited similar levels, and there was no significant difference between the ABX and ABX+Pae groups (Figure 6B). In addition, the aortic tissue from the FM(Pae) group showed less mRNA expression levels of IL-1β, TNF-α, IL-6, and IL-17A but higher IL-10 compared with the aortic tissue from the FM(HFD) group (Figure 6C). In general, Pae significantly downregulated the level of proinflammatory cytokines as well as upregulated the immunosuppressive cytokines in AS mice.

### LOX Upregulation Links Microbial Changes to Progression of Vascular Fibrosis

**FIGURE 7:**
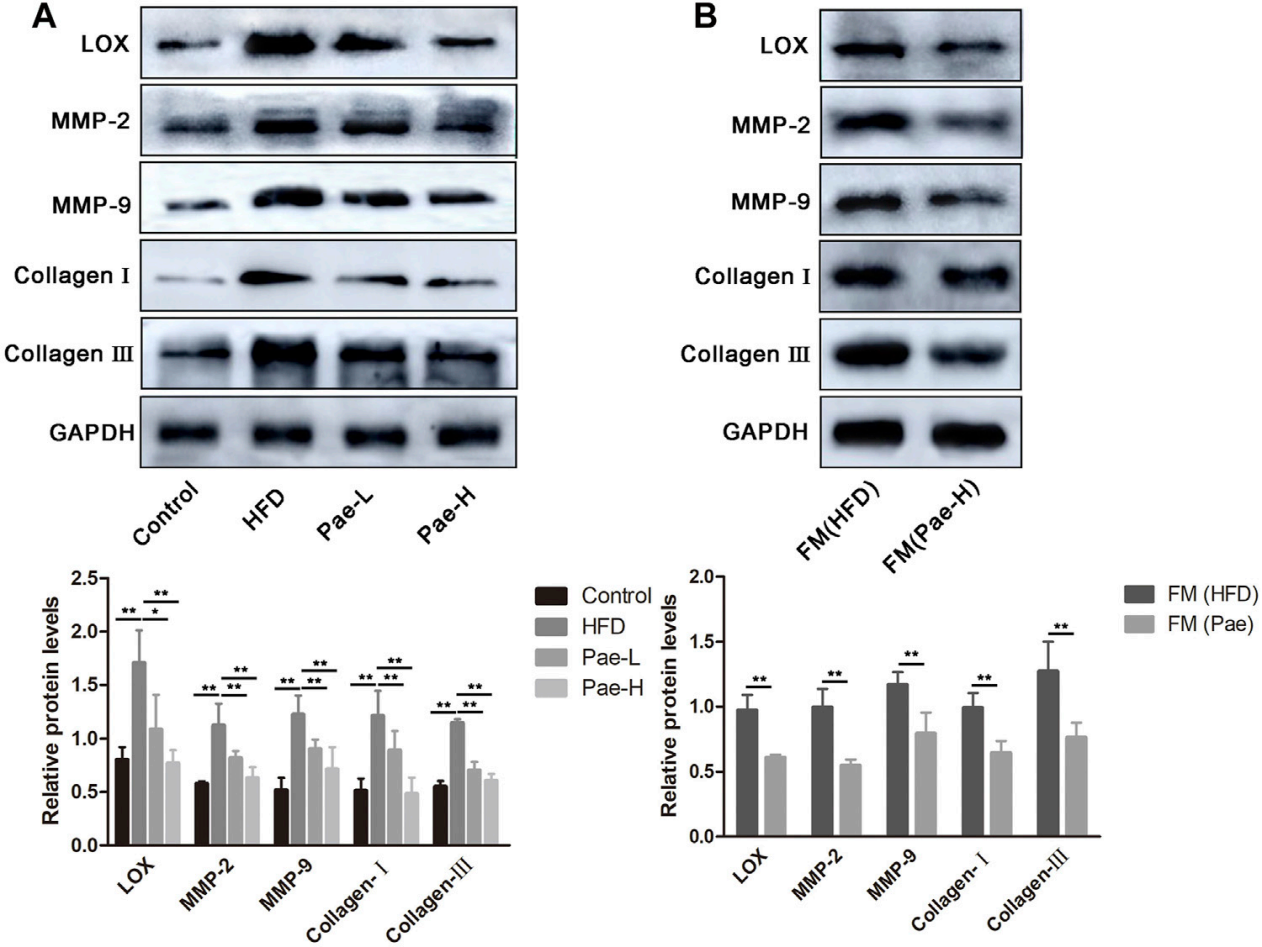
Pae inhibited LOX expression and vascular fibrosis of in a gut microbiota- dependent manner in ApoE^-/-^ mice. **(A)** The expressions of LOX and fibrosis-related indicators (MMP-2/9 and collagen I/III) were examined by western blot in control, HFD, and Pae groups. **(B)** The expressions of LOX and fibrosis-related indicators (MMP-2/9 and collagen I/III) were examined by western blot in FM(HFD) and FM(Pae) groups. Data are presented as mean ± SEM, *n* = 3. ^*^ *p* < 0.05, ^**^ *p* < 0.01.

To further determine how Treg/Th17 balance affected vascular fibrosis, we analyzed the protein expression of LOX and fibrosis-related indicators and found LOX and fibrosis-related indicators (MMP-2/9 and collagen I/III) as highly upregulated expression in aortas of AS mice (Figure 7A). LOX has been implicated in various fibrotic conditions and plays a role in AS. IL-17, a marker of Th17 cells, promotes LOX expression by activating the ERK1/2-AP-1 pathway. However, the production of proinflammatory cytokines is not always profibrotic. Specifically, IL-10 secreted by Treg cells can resist LOX-mediated fibrosis. As shown in Figure 7A, with the treatment of Pae and compared to the HFD group, it could be found that the expressions of LOX, MMP-2/9, and collagen I/III were downregulated significantly. In addition, the aortic tissue from the FM(Pae) group showed downregulated protein expression levels of LOX, MMP-2/9, and collagen I/III compared with the aortic tissue from the FM(HFD) group (Figure 7B). Collectively, these results indicated that the balance of Treg/Th17 regulated by Pae downregulated the expression of LOX and inhibited vascular fibrosis.

## Discussion

AS is a chronic inflammatory disease characterized by the accumulation of lipids, vascular fibrosis, and inflammation. Our previous studies have demonstrated that Pae has a certain anti-AS effect *in vivo* and *in vitro* (Li et al., 2009; Pan and Dai, 2009). In addition, Pae delays the progression of renal fibrosis and pulmonary fibrosis (Liu et al., 2017; Zhang et al., 2018). Therefore, we first studied whether Pae has anti-AS and antivascular fibrosis effects *in vivo*. As expected, significant reduction of the formation of the aortic plaque was achieved with Pae treatment. Furthermore, Pae decreased the protein expression of α-SMA, a marker of vascular fibrosis, and ameliorated vascular collagen deposition in AS mice.

Mounting evidence from animal and human studies supports the fact that gut microbiota can influence the occurrence and development of AS (Zhu et al., 2020). Therefore, it was necessary to study whether gut microbiota participate in the antivascular fibrosis effect of Pae on AS. It was worth noting that the vascular fibrosis and collagen deposition between ABX and ABX+Pae groups displayed no significant difference. Microbiota transfer from Pae-treated donors reduced the formation of aortic plaque, and collagen deposition was reduced in the recipients, suggesting that a particular constellation of microbial compounds, available in Pae-treated mice, was capable of affecting the process of vascular fibrosis.

Recently, gut microbiota has been thought to be the main factor that provokes the immune system (Pickard et al., 2017), and the disturbance of the microbiota–host relationship is closely linked to AS (Kurilshikov et al., 2019). AS patients showed reduced gut microbiota biodiversity, including a lower abundance of SCFA-producing beneficial commensal bacteria (Verhaar et al., 2020). In agreement, we found that Pae treatment markedly transformed the biological community structures. Gut microbiota-derived SCFAs play an important role in Tregs induction in inflammatory disease (Xu et al., 2017). We found a significant increment of the SCFA level in cecal contents and serum of AS mice, which correlated with a high amount of gut bacteria including *Bacteroidia*, *Clostridia*, *Clostridium Ⅳ*, Lachnospiraceae, and *Lactobacillus* known to contribute to SCFA production (Chen et al., 2020; Zhang et al., 2021). The data obtained from the analysis of gut microbiota in mice might be highly relevant to humans, who are much more diverse in microbes, but with similar metabolic characteristics.

Gut microbiota-derived SCFA has recently been implicated in promoting the development of Treg cells and possibly other T cells (Liu et al., 2020), and protects against HFD-induced AS mice (Li et al., 2018). The Treg/Th17 is a pair of new balance that differs from Th1/Th2 balance in the immune system, which is a critical intervention target during the development process of AS. Previous studies have shown that Pae can reduce host inflammation by regulating helper T cell subsets (Meng et al., 2019). Our data revealed that the regulation effect of Pae on the balance of Treg/Th17 may be related to gut microbiota, as the ratio of Treg/Th17 balance displayed no significant difference between the ABX and ABX+Pae groups. Furthermore, microbiota transfer from Pae-treated donors corrected Treg/Th17 balance in the recipients, supporting the hypothesis that Pae has an important microbiome-regulatory role in AS.

Chronic inflammation is the key factor in AS pathogenesis, while inhibiting inflammatory response controls AS evolution. Treg cells have been identified as dedicated suppressors of inflammation through the secretion of the anti-inflammatory cytokine IL-10 and the inhibition of Th17 cells (He et al., 2020). Therefore, correcting the balance of Treg/Th17 is essential to maintaining the level of inflammatory factors. Our data revealed a reduced level of proinflammatory cytokines (IL-1β, IL-6, TNF-α, and IL-17) as well as upregulation of the anti-inflammatory cytokine IL-10 in AS mice after Pae treatment. Moreover, bacteria removal and FMT experiments have also confirmed a linear connection between Pae, gut microbiota, and Treg/Th17 balance.

As mentioned above, Pae had an effect on restoring the balance of Th17/Treg and relative inflammatory cytokines in a gut microbiota-dependent manner. Here, we need to bring up another important issue. Recently, Treg and Th17 cells are important in promoting fibrosis (Lu et al., 2020). Identifying the mechanism underlying the effects of Pae on Treg/Th17 balance mediated vascular fibrosis would aid the antifibrotic therapy development. Emerging evidence suggests that the reciprocal modulation of Th17 and Treg on myocardial fibrosis was mediated by reciprocal regulation of LOX expression (Lu et al., 2020). However, it is unclear whether LOX is involved in vascular fibrosis. Our findings elucidated that the expression of LOX and fibrosis-related indicators (MMP-2/9 and collagen I/III) was downregulated after Pae treatment. Important mechanism of Pae in the control of vascular fibrosis downstream of microbial alterations was illuminated by antibiotic and FMT experiment.

**FIGURE 8:**
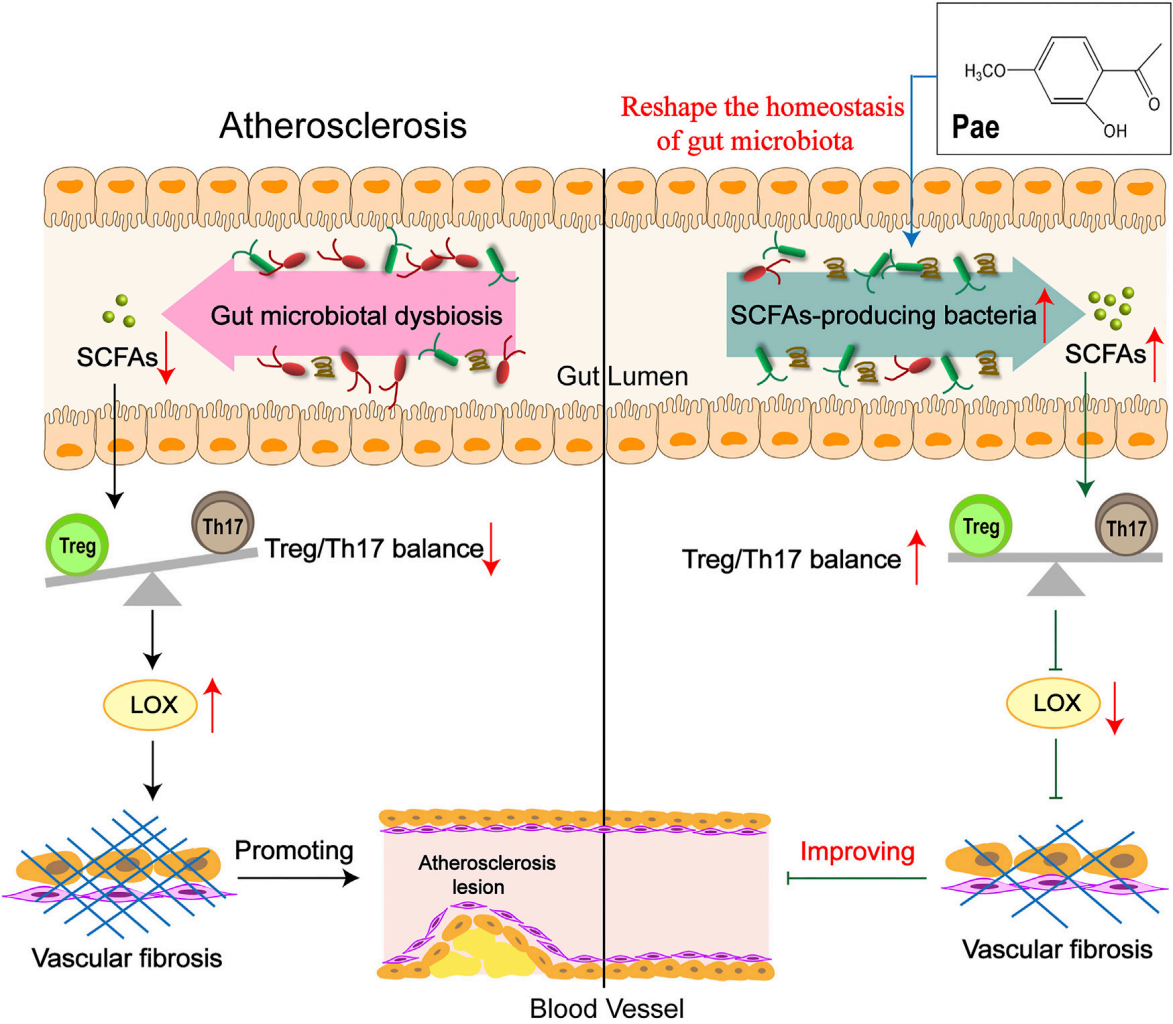
Schematic illustration of Pae attenuated vascular fibrosis through regulating Treg/Th17 balance in a gut microbiota-dependent manner. The gut microbiota of AS mice was disordered, causing the balance of Treg/Th17 cells to tilt toward Th17 cells, inducing the increase in LOX expression in the aorta, thereby promoting vascular fibrosis. Pae can increase the abundance of short-chain fatty acid-producing bacteria to directly upregulate SCFA levels in AS mice, leading to the decrease in LOX expression by restoring the Treg/Th17 balance, thereby inhibiting vascular fibrosis, which was an important mechanism of Pae in improving vascular fibrosis.

In conclusion, our data suggest a model in which treatment of Pae results in alterations in the SCFA-produced microbiota, which, coupled with increased SCFAs and corrected Treg/Th17 balance, leads to upregulated LOX expression, alleviating vascular fibrosis and AS, particularly in a microbiota-dependent manner (Figure 8). These findings provide compelling evidence that Pae may be used as a prebiotic agent to treat AS, which is expected to improve the understanding of the pathogenesis of vascular fibrosis.

## Data Availability

The raw data supporting the conclusion of this article will be made available by the authors, without undue reservation.
